# Formulation and *ex vivo* characterization of a kaempferol transethosomal hydrogel for the prevention of UV-induced cutaneous damage

**DOI:** 10.3389/fbioe.2026.1963532

**Published:** 2026-10-12

**Authors:** Federico Santamaria, Andrea Galvan, Nicholas Castaldini, Valentyn Dzyhovskyi, Paolo Mariani, Alessia Pepe, Maria Grazia Ortore, Michal Rawski, Debora Santonocito, Mascia Benedusi, Nicolas Huang, Elisa Marlot, Giuseppe Valacchi, Manuela Malatesta, Laura Calderan, Elisabetta Esposito

**Affiliations:** 1 Department of Chemical, Pharmaceutical and Agricultural Sciences, University of Ferrara, Ferrara, Italy; 2 Department of Neurosciences, Biomedicine and Movement Sciences, University of Verona, Verona, Italy; 3 Department of Life and Environmental Sciences, Polytechnic University of Marche, Ancona, Italy; 4 Department of Life Science, Health and Health Professions, Link Campus University, Roma, Italy; 5 SOLARIS, National Synchrotron Radiation Centre, Jagiellonian University, Kraków, Poland; 6 Department of Drug and Health Sciences, University of Catania, Catania, Italy; 7 Department of Neurosciences and Rehabilitation, University of Ferrara, Ferrara, Italy; 8 Université Paris-Saclay, CNRS, Institut Galien Paris-Saclay, Orsay, France; 9 Department of Environmental Sciences and Prevention, University of Ferrara, Ferrara, Italy; 10 Animal Science Department, Plants for Human Health Institute, NC Research Campus, NC State University, Kannapolis, NC, United States; 11 Department of Food and Nutrition, Kyung Hee University, Seoul, Republic of Korea

**Keywords:** ethosome, kaempferol, pluronic gel, skin, transethosome

## Abstract

In this study, lipid nanovesicular systems such as ethosomes and transethosomes dispersions were studied as potential vehicles for the cutaneous administration of kaempferol, a flavonol with antioxidant and chemopreventive properties. The lipophilicity of kaempferol requires specialized delivery systems suitable for transdermal administration. Kaempferol loading in ethosomes and transethosomes, prepared by ethanol injection methods, was investigated. The morphology of the vesicles was studied by cryogenic electron microscopy and by small angle X-ray scattering, while the size distribution was assessed by photon correlation spectroscopy. To make transethosome dispersions suitable for cutaneous application, Pluronic F-127 was used as a gelling agent. The physicochemical stability of the formulations was evaluated over a period of 3 months. To study the effect of the applied formulations on the skin, spreadability and leakage tests were performed. In vitro permeation studies were conducted using Franz diffusion cells and HPLC, to evaluate the diffusion kinetics of Kaempferol from the formulations. The evaluation of the dispersion macroscopic stability enabled to select transethosomes, loaded with 0.25 mg/ml of kaempferol. The results showed a promising 96% entrapment capacity, corresponding to 0.24 mg/ml and physicochemical stability of transethosomes, both in terms of size distribution and kaempferol content. Quantitative analysis via HPLC revealed that the drug diffuses more slowly from Pluronic-containing lipid formulations. Conversely, enhanced permeation through the membrane was observed for transethosomes. The *ex vivo* study on human skin explants demonstrated that the kaempferol-loaded transethosomal gel is able to limit the oxidative damage induced by UV exposure.

## 1 Introduction

Lipid-based systems have attracted considerable attention in pharmaceutics owing to their exceptional drug delivery capabilities (Limongi et al., 2021). Crucially, the spontaneous self-assembly of natural lipids, most notably phosphatidylcholine (PC), yields bilayered vesicles whose supramolecular architecture closely mimics physiological membranes (Phapal et al., 2017). These nanovesicles, comprising liposomes, transferosomes, ethosomes (ET), and transethosomes (TE), are highly effective at accommodating and delivering both hydrophilic and lipophilic therapeutic agents (Nayak et al., 2022; Batur et al., 2024). Although the utility of liposomes as drug delivery systems has been widely established since the 1960 for controlling drug release across various administration routes (Gunda et al., 2023), their efficacy in transdermal delivery is often restricted by an inability to penetrate the deeper cutaneous layers (Pierre and Dos Santos Miranda Costa, 2011; Souto et al., 2021). Specifically, some studies suggest that these conventional vesicles remain confined to the superficial skin layers, where their affinity for *stratum corneum* lipids prompts fusion and the subsequent formation of a depot that gradually releases the active compounds (Pollet et al., 2021; Akombaetwa et al., 2023; Huntjens et al., 2023). Alternatively, other researchers have demonstrated that intact liposomes can only access deeper tissue via skin appendages, such as hair follicles and sweat glands (El Maghraby et al., 2006; Pollet et al., 2021).

To overcome the penetration limitations typical of conventional liposomes, Touitou et al. introduced ET (Touitou et al., 2000). The primary distinction in their composition is the inclusion of a high concentration of ethanol (20%–45%) alongside phospholipids and water (Almuqbil and Aldhubiab, 2025). This ethanolic component imparts several distinct advantages, namely,: (i) reduction of vesicle size, (ii) enhancement of vesicle stability and deformability compared to classic liposomes, (iii) improvement of the entrapment efficiency of lipophilic drugs, and (iv) issue of a negative surface charge that prevents vesicle aggregation and subsequent drug leakage (Almuqbil and Aldhubiab, 2025). Furthermore, ethanol acts as a potent penetration enhancer, fluidizing skin lipid matrix to promote vesicle migration into the deepest dermal layers (Godin and Touitou, 2003). To build upon this technology and further amplify nanovesicle permeability, Song et al. developed TE in 2012 as a second-generation ET system (Song et al., 2012). TE incorporate edge activators, such as glycerol or surfactants, which intercalate into the lipid bilayer. By disrupting the lipid packing, these agents modulate vesicle size while significantly increasing bilayer flexibility and permeability, ultimately rendering TE even more malleable and penetrative than conventional ET (El Zaafarany et al., 2010; Shinde and Somani, 2023).

Kaempferol (3, 5, 7-trihydroxy-2-(4-hydroxyphenyl)-4H-1-benzopyran-4-one, KF) (Supplementary Figure S1) is a widespread natural flavonol abundantly present in various dietary and botanical sources, including beans, bee pollen, broccoli, cabbage, capers, cauliflower, chia seeds, chives, cumin, moringa leaves, endive, fennel, garlic, tea, kale, and Ginkgo biloba. Over the past few decades, KF has attracted substantial research interest due to its diverse and potent pharmacological profile, which encompasses antioxidant, anti-inflammatory, antimicrobial, antitumor, cardioprotective, neuroprotective, antidiabetic, and photoprotective activities (Imran et al., 2019; Sekiguchi et al., 2019; Kurek-Górecka et al., 2020; Shahbaz et al., 2023; Alrumaihi et al., 2024). Its capacity to mitigate oxidative stress and downregulate pro-inflammatory mediators underscores its potential as a therapeutic candidate for various skin conditions. Specifically, KF is highly relevant in: (i) mitigating photoaging, by counteracting UV-induced oxidative damage and inhibiting matrix metalloproteinases (MMPs); (ii) accelerating wound healing, through the promotion of re-epithelialization and collagen synthesis; and (iii) managing inflammatory dermatoses, by suppressing localized immune responses and reducing oedema (Permana et al., 2020; Mostafa et al., 2021).

KF mitigates UV-induced oxidative stress and inflammatory damage by targeting lipid peroxidation products and matrix-degrading enzymes. Ultraviolet radiation triggers the excessive accumulation of 4-hydroxynonenal (4-HNE), a toxic byproduct of lipid peroxidation that damages cellular proteins and drives inflammatory cascades. By directly scavenging reactive oxygen species and reinforcing internal antioxidant defenses, KF reduces 4-HNE accumulation and prevents its downstream toxic signaling (Jang et al., 2011). This suppression of oxidative stress concurrently blocks the activation of pro-inflammatory pathways (such as the nuclear factor kappa-light-chain-enhancer of activated B cells, NF-*K*B), which in turn prevents the upregulation and release of MMPs (Lim and Kim, 2007). By suppressing MMPs, KF inhibits the enzymatic breakdown of collagen and extracellular matrix components, offering protection against UV-induced acute photo-inflammation and dermal photoaging (Wang et al., 2020).

However, despite these therapeutic and protective properties, the clinical translation of KF is heavily restricted by its high lipophilicity, which results in negligible aqueous solubility, poor permeability, and consequently, limited local bioavailability. Furthermore, the *stratum corneum* acts as a formidable barrier that severely impedes the passive diffusion of KF, hindering the achievement of therapeutically effective concentrations within the deeper epidermal and dermal layers. To overcome these biopharmaceutical hurdles, advanced nano-delivery systems have emerged as a promising strategy to enhance the solubility, stability, and cutaneous permeation of the flavonol. To this end, several researchers have successfully encapsulated KF within nanostructured matrices, such as liposomes, phytosomes, and solid lipid nanoparticles, to optimize its physicochemical stability, improve apparent solubility, and provide controlled release kinetics for the targeted treatment of skin disorders (Permana et al., 2020; Mostafa et al., 2021; Fang et al., 2025; 2026; Siddhi and Mrityunjaya, 2026).

In the present investigation, a preformulative study was conducted to select a nanovesicular system for the transdermal delivery of KF. ET and TE were prepared using ethanol injection method. The resulting colloidal dispersions were characterized by size, zeta potential, and morphology, followed by an evaluation of their entrapment capacity and *in vitro* KF permeation. To facilitate topical application and prolonged skin contact, a TE gel was formulated using the block copolymer Poloxamer and evaluated for leakage, spreadability, and rheological behaviour. Moreover, Small-Angle X-ray Scattering (SAXS) was employed to investigate the supramolecular organization of the formulations. Finally, *ex vivo* investigations on human skin explants were performed to confirm the efficacy of the selected TE_KF_-gels in mitigating the oxidative stress induced by UV exposure.

## 2 Materials and methods

### 2.1 Materials

Kaempferol (KF), the copolymer poly (ethylene glycol)-block-poly (propylene glycol)-block-poly (ethylene glycol) Pluronic F127 (PEO98-POP67-PEO98, POL), Tween 80 (polyoxyethylene sorbitan monooleate, TW80), and STRAT-M® membranes were purchased from Merck Life Science S. r.l. (Milan, Italy). The soybean lecithin (PC) (90% phosphatidylcholine) was Epikuron 200 from Lucas Meyer (Hamburg, Germany). All the solvents employed in this study where HPLC grade, while all the other chemicals were analytical grade.

### 2.2 Preparation of ethosomes and transethosomes

ET and TE were prepared by a cold method, starting with the preparation of a PC solution. Briefly, PC was solubilized directly in ethanol at room temperature under magnetic stirring (IKA RCT basic, IKA®-Werke® GmbH and Co. KG, Staufen, Germany) to achieve a final concentration of 30 mg/mL. For TE preparation, TW80 was additionally solubilized in the PC-ethanol solution at room temperature, reaching a final concentration of 10 mg/mL.

Subsequently, the PC-ethanol solution, enriched with TW80 in the case of TE, was added dropwise into bidistilled water, chosen to ensure chemical and microbiological purity, kept under magnetic stirring at 750 rpm. Afterwards, magnetic stirring was maintained for 30 min to allow proper vesicle formation. The final ethanol/water ratio was 30:70 (v/v) for both ET and TE.

KF-loaded formulations (ET_KF_ and TE_KF_) (0.2–0.5 mg/mL) were prepared by prior dissolution of the drug in the ethanol solution, with the entire preparation process performed in dark conditions.

### 2.3 Cryogenic electron microscopy (cryo-em)

Cryo-EM data collection was performed at the National Cryo-EM Centre SOLARIS (Kraków, Poland) using a Titan Krios G3i microscope (Thermo Fisher Scientific, Krakow, Poland) operated at 300 kV. Images were acquired at a nominal magnification of × 105,000, yielding a pixel size of 1.7 Å, utilizing EPU 2.10 software. Movie stacks were recorded on a Gatan K3 direct electron detector paired with a BioQuantum imaging filter operating in counting mode with a 20-eV slit width. The total electron dose was 41.09 e−/Å^-2^ with a targeted defocus of −2.0 µm. Sample vitrification was achieved using a Vitrobot mkIV (Thermo Fisher Scientific, Krakow, Poland) at 4 °C and 95% humidity. Grids were prepared by applying 3 µL of sample suspension, followed by a 3-s double-sided blot at a force of 3, before being plunge-frozen into liquid ethane.

### 2.4 Photon correlation spectroscopy (PCS)

PCS technique was employed to measure the size distribution of the nano-sized formulations, evaluating the values of Z-Average, corresponding to the mean hydrodynamic diameter, and the polydispersity indexes (PI). Particularly, a Zetasizer Pro (Malvern Instr., Malvern, England) was employed for the analyses. The instrument is equipped with a 5 mW helium neon laser, producing a wavelength output of 633 nm. The analyses were conducted at 25 °C with a 173° detection angle and 120 s equilibration time. The samples were diluted 1:10 (v/v) with bi-distilled water. Size distribution was obtained by the “CONTIN” method (Pecora, 2000), then analysed using ZSXplorer software. Each analysis was carried out in triplicate from which the mean is derived.

### 2.5 Zeta potential

Zeta potential was evaluated using Zetasizer Pro (Malvern Instr., Malvern, England). This technique involves the measurement of scattering of incident laser light by moving particles according to their electrophoretic mobility. The instrument is equipped with a 5 mW helium neon laser that produces a wavelength output of 632.28 nm. The analyses were conducted at 25 °C. The samples were diluted 1:10 (v/v) with bidistilled water inside plastic tube and then put inside cuvette DTS1070 (Malvern Instr., Malvern, England).

### 2.6 Entrapment capacity

The entrapment capacity (EC) of KF was quantified via ultrafiltration (Bondi et al., 2025), separating the nanocarriers to analyse the associated drug using HPLC. One day after preparation, 500 µL of the colloidal system was centrifuged at 4,000 rpm for 20 min using Amicon Ultra centrifugal filters (NMWCO 10 kDa, Merck Millipore, Cork, Ireland) in a Spectrafuge™ 24D microcentrifuge (Infitek Inc.). The isolated retentate was diluted in ethanol (1:10 v/v) and magnetically stirred for 30 min to lyse the nanostructures. To determine the total formulation drug content, a 100 µL aliquot of the whole dispersion was processed under identical conditions. All disrupted samples were filtered through 0.22 µm nylon syringe filters before HPLC injection.

Finally, the entrapment capacity (EC), was calculated as:

$$\text{EC}=D/T_{D}\times 100$$

In the equation, D represents the amount of drug retained by the vesicles, and T_D_ refers to the real drug content in the entire formulation.

### 2.7 HPLC analysis

HPLC analysis was performed using a Perkin Elmer series 200 system (United States) equipped with a UV detector set at 360 nm. Chromatographic separation was achieved on a stainless-steel BDS Hypersil C-18 reversed-phase column (150 × 4.6 mm) packed with 5 μm particles (Thermo Fisher Scientific, Rodano, Italy). The mobile phase consisted of a methanol/water mixture (80:20 v/v) plus 0, 1% v/v of trifluoroacetic acid, pumped at a flow rate of 1 mL/min.

### 2.8 pH measurement

The measurement of pH was carried out with a Bench Meter series 50 Violab pHmeter (XS Instruments, Modena, Italy), equipped with an Electrochemical Sensor. All the measurements were done in triplicate on independent experiments.

### 2.9 Stability studies

The physicochemical stability of the formulations was evaluated by monitoring changes in particle size distribution, zeta-potential, and KF loading during storage at 4 °C in the dark over a 3-month period. Measurements were performed at predetermined time intervals. Size distribution and zeta-potential were analysed using a Zetasizer Pro, while KF chemical stability was quantified via HPLC.

### 2.10 *In vitro* permeation test (IVPT)

*In vitro* permeation tests (IVPT) were conducted using vertical Franz diffusion cells (0.9 cm orifice diameter; PermeGear Inc., Hellertown, PA, United States) equipped with Strat-M® synthetic membranes. Strat-M® consists of two polyethersulfone layers atop a polyolefin bottom layer, impregnated with synthetic lipids; this specific architecture effectively mimics the barrier properties of the *stratum corneum* (Bondi et al., 2025). The receptor compartment was filled with 5 mL of an ethanol/water mixture (50:50 v/v) to maintain sink conditions, due to KF lipophilic character, and to ensure detectable HPLC analyte concentration. This receiving phase was maintained at 32 °C under continuous magnetic stirring at 500 rpm throughout the experiment. The donor compartment was loaded with 1 mL of the formulation and sealed to prevent evaporation. Aliquots of the receiving phase (500 µL) were sampled hourly over 24 h and analysed by HPLC, with each withdrawn volume immediately replaced with an equal volume of fresh receptor medium.

For data analysis, the cumulative amount of permeated drug per unit area μg/cm^2^ was plotted against time. Under sink conditions, the drug concentration in the receptor compartment is negligible compared to the donor compartment, aligning with Fick’s law where the concentration gradient drives diffusion. The steady-state flux (J_ss_, μg cm^-2^ h^-1^). Drug permeability coefficients “K_p_” value can be calculated from J_ss_ according to the equation:

$$K_{p}=J_{\text{ss}}/C_{d}$$

where C_d_ corresponds to the drug concentration in the donor phase.

Lag time (“T_lag_”), which is the time needed to reach steady-state conditions, could be obtained from the intersection of the line with the x-axis.

### 2.11 Gel preparation

TE vesicular dispersions were thickened using POL. Precisely, 1 day after preparation, TE and TE_KF_ were thickened by direct addition of POL to the dispersion kept in an ice bath under magnetic stirring. To avoid lump formation, the addition was performed gradually, maintaining the stirring until the system became uniform. Three different concentrations of POL were evaluated (i.e. 15, 18% and 20% w/w). Spreadability and leakage of TE gels (hereafter referred to as TE-PG) were evaluated 1 day after preparation.

### 2.12 Spreadability assay

The spreading capacity of the TE-PG was determined at room temperature (25 °C), 24 h post-preparation, following the method described by (Sicurella et al., 2021). Briefly, a 100 mg gel sample was placed at the centre of a 3 cm diameter Petri dish. A constant pressure was then applied by placing a glass dish loaded with a 50 g mass on top of the sample. After 10 s, the diameter of the area covered by the spread gel was measured. The analysis was performed in triplicate, and results were expressed as mean values ± standard deviation using the following equation:

S=m x l / t

where: “S” represents the spreadability of the gel formulation, “m” is the mass loaded onto the upper plate (50 g), “l” is the diameter of the spread gel area (cm), and “t” is the exposure time (10 s).

### 2.13 Leakage assay

To evaluate the leakage behaviour of the formulations, an agar gel substrate was prepared in-house by dissolving 1.5% w/w agar in a phosphate buffer (pH 5.5) under stirring at 95 °C until complete solubilization (Sicurella et al., 2021). For visual tracking, the semisolid formulations were dyed (0.05% w/w) using acid yellow 25, oil red, or Coomassie blue.

For the assay, a 50 mg sample of the coloured formulation was applied to one end of an agar slide secured within a Petri dish. The dish was then positioned vertically at a 90° angle against the inner wall of a transparent chamber maintained at 32 °C ± 1 °C. The downward migration distance along the slide was recorded 10 s after sample placement (Supplementary Figure S2). Gel leakage was quantified as a percentage based on the difference between the total length of the agar slide and the distance travelled by the gel. All measurements were conducted in triplicate, and the data were reported as mean values ± standard deviation.

### 2.14 Rheological analysis

Rheological measurements were performed on TE-PG, loaded or not with KF (TE_KF_-PG and TE-PG, respectively), with a controlled-stress rotational rheometer (AR-G2 or HR10, TA Instruments, New Castle, DE, United States). The geometry used was an aluminium cone–plate with a diameter of 40 mm, an angle of 1° and a truncation of 28 μm, equipped with a solvent trap to avoid evaporation. Flow ramp and oscillation amplitude sweep were performed at 20 °C. For flow ramps, the shear rate was varied from 0.01 to 1,000 s^−1^ in 180 s, then was fixed at 1,000 s^−1^ during 60 s, and finally was varied from 1,000 to 0.01 s^−1^. For oscillation amplitude sweeps, the deformation amplitude increased from 0.01% to 1,000% at a fixed frequency of 1 Hz. Oscillation temperature ramps from 5 °C to 50 °C were performed at a rate of 1 °C/min with constant deformation in the linear regime and were controlled by a Peltier plate. Before starting the experiments, a 3-min conditioning time at either 20 °C (for flow ramps and oscillation amplitude sweeps) or 5 °C (for oscillation temperature ramps) was applied. All experiments were performed in triplicate for reproducibility.

### 2.15 Small angle x-ray scattering (SAXS)

SAXS experiments were performed at the Austro-SAXS beamline of Elettra Synchrotron (Trieste, Italy). The samples were put in a quartz capillary with a diameter of 1.5 mm and measured at 25 °C and 32 °C within a KPR (Peltier heating/cooling) sample holder (Anton Paar, Graz, Austria). For each sample, SAXS measurements were performed with a 10 s exposure time, acquiring 10 consecutive frames. Two-dimensional (2D) data were corrected for background and detector efficiency, and then radially averaged to derive the scattered intensity I(q) as a function of the modulus of the scattering vector q = 4π sinθ/λ (2θ is the scattering angle and λ the X-ray wavelength). The sample-to-detector distance provided a q-range between 0.1 and 4 nm^-1^. The detector used for the measurements was a 2D Pilatus3 1M Detector System with a pixel size of 172 × 172 μm^2^ and a discrete energy of 8 keV (corresponding to the beam wavelength of 0.154 nm).

### 2.16 *Ex vivo* test of kaempferol-loaded transethosome gel on human skin explants

The protective potential of TE_KF_-PG against the oxidative damage induced by UV exposure was tested *ex vivo* on human skin explants derived from excess tissue obtained from a 39- and 40-year-old Caucasian women submitted to abdominoplasty surgery.

After a brief rinse of the skin explants in sterile phosphate buffered saline (PBS), circular samples with a diameter of 1.2 cm were excised and placed in multiwell plates containing 2 mL of DMEM medium supplemented with 10% FBS, 1% glutamine, and 1% streptomycin. The upper part of the epidermis was exposed to the air establishing the air-liquid interface (ALI) setup. After incubating overnight at 37 °C with 5% CO_2_, the following formulations were applied on the *stratum corneum* of some skin explants: a suspension of kaempferol in ethanol/water 30:70 v/v (SUSP_KF_), TE, TE_KF_, and TE_KF_-PG. Twenty-four h after formulation application, skin samples were exposed to 200 mJ UVA/UVB light as reported in (Ferrara et al., 2020) for 20 s, mimicking an 8 hour-exposure to the sun. Some skin samples were exposed to UV but were not treated with any formulation. Skin samples that did not receive any UV or formulation treatment served as control (CT). The culture medium was refreshed every 24 h, and the UV exposure and formulation application were repeated accordingly. Skin samples were harvested at two distinct time points: at Day 1, which involved a single UV exposure and formulation application, and at Day 3, which involved three consecutive daily applications of UV and formulations. At each time point, skin explants were fixed in 10% neutral buffered formalin, dehydrated in ethanol and embedded in paraffin wax.

Deparaffinized and rehydrated skin sample sections (5 µm in thickness) were then processed for immunofluorescence analysis of 4-HNE and type I collagen (COL I). An anti-4-HNE Polyclonal Antibody (Chemicon®, Sigma-Aldrich, Catalog Number #AB5605) was used at 1:500 dilution in PBS and revealed with an anti-goat secondary antibody conjugated with Alexa Fluor™ 568 (Invitrogen Thermo Fisher Scientific, Waltham, MA, United States, Catalog #A11057) and diluted at 1:1,000 in PBS. An anti-Collagen I Monoclonal Antibody (EPR7785, Abcam, Catalog Number #ab138492) was used at 1:2000 dilution in PBS and revealed with an anti-rabbit secondary antibody conjugated with Alexa Fluor™ 488 (Invitrogen Thermo Fisher Scientific, Catalog #A11055) and diluted at 1:1,000 in PBS. Skin samples were observed with a ZEISS LSM 710 confocal microscope (Carl Zeiss Microscopy GmbH, Jena, Germany).

For quantitative analyses of immunofluorescence, 36 skin images (40X magnification) per condition (18 from donor 1 and 18 from donor 2) of the epidermal (for 4-HNE) and the dermal (for COL I) compartment were considered. The fluorescence intensity of immunolabelling was measured with ImageJ (United States National Institutes of Health, Bethesda, MD, United States) and calculated for each sample as the percentage of the respective control.

For statistical analysis, the Shapiro-Wilk test was applied to verify the normal distribution of data. Since data samples revealed a non-normal distribution, the non-parametric Mann Whitney test for was used pairwise comparisons. Data are presented as mean values ± standard error (SE), and significance was set at p ≤ 0.05.

## 3 Results

### 3.1 Preparation of ethosomes and transethosomes

A preformulation study was carried out to identify an appropriate KF-loaded nanosystem. Both ET and TE were prepared via the EI method (Bondi et al., 2025) according to compositions previously established by our research group (Sguizzato et al., 2021; Ferrara et al., 2022). For the TE formulation, the non-ionic surfactant TW80 was incorporated as an edge activator (Bnyan et al., 2018); complete compositional details are provided in Table 1.

**TABLE 1:** Composition of ET and TE.

Nanosystem	PC^a^(%, w/w)	Ethanol (%, w/w)	TW80^b^(% w/w)	Water (%, w/w)	KF^c^(% w/w)	Aspect
ET	0.9	29.100	-	70	-	Homogeneous
ET_KF_	0.9	29.080	-	70	0.025	Phase separation
TE	0.9	28.800	0.3	70	-	Homogeneous
TE_KF_	0.9	28.775	0.3	70	0.025	Homogeneous

^a^ Phosphatidylcholine.

^b^ Tween 80.

^c^ kaempferol.

Every blank formulation appeared homogeneous and showed no signs of phase separation, although the TE formulations were visibly more translucent. On the other hand, loading KF (0.25 mg/mL) into the ET forms consistently caused phase separation. Conversely, loading KF into the TE forms produced no phase separation, suggesting that TW80 likely provides stabilization and improves KF solubilization, preventing disruption of phospholipid packing that causes vesicle rupture and phase separation in ET. Consequently, both ET and ET_KF_ were omitted from further investigation. The size distribution and zeta potential of the TE formulations, recorded 24 h after preparation, are presented in Table 2.

**TABLE 2:** Physicochemical characterization of transethosomes.

Formulation	Z-Average (nm) ± s.d.	PI^a^ ± s.d	Z-potential (mv) ± s.d.	pH ± s.d	EC^b^ (%)
TE	107.50 ± 1.82	0.13 ± 0.01	−23.80 ± 6.34	6.56 ± 0.06	-
TE_KF_	108.33 ± 3.85	0.14 ± 0.03	−20.21 ± 1.47	5.22 ± 0.12	96.44 ± 0.02

SD, Standard Deviation.

^a^ Polydispersity index.

^b^ entrapment capacity; data are the mean of 4 independent determinations on different batches.

TE exhibited a mean diameter of roughly 110 nm and homogeneous size distribution, as confirmed by polydispersity index values consistently below 0.2. Notably, the inclusion of KF did not alter the vesicle Z-Average or PDI (Table 2). Zeta potential values, approaching −20 mV, indicate sufficient electrostatic repulsion between vesicles to prevent aggregation, thereby ensuring the long-term physicochemical stability of the formulation. The distinct negative surface charge of the vesicles is attributable to the presence of EtOH and the hydroxyl groups of Tween 80 (Bin Jardan et al., 2023). Upon drug loading (TE_KF_) a slight shift in Zeta potential was observed. Since both formulations contain equivalent amounts of ethanol and TW80, this change in surface charge is directly associated with the association and entrapment of KF into the lipid bilayer, where its phenolic hydroxyl groups can interact with the phospholipid headgroups at the vesicle interface, thereby altering the charge distribution at the slip plane. The pH values were compatible with skin administration (pH 4–6 (Lukić et al., 2021)), with slightly more acidic values in the case of TE_KF_. HPLC analysis was used to determine both the total KF content in the formulation and the EC, which represents the fraction of KF successfully incorporated into the TE. The high EC value indicates that nearly all the drug is associated with the vesicles, as expected given its lipophilic nature. Finally, cryo-EM analysis provided detailed insights into the structural morphology of both TE and TE_KF_ (Figure 1).

**FIGURE 1:**
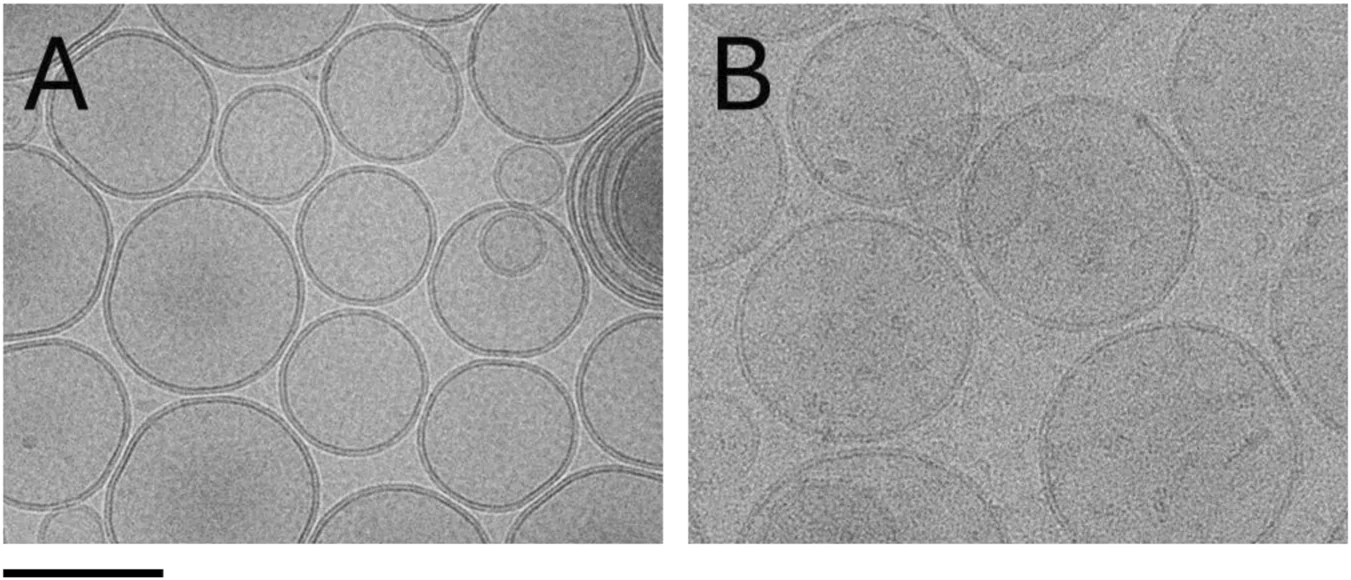
Cryo-EM images of TE **(A)**, and TE_KF_ **(B)**. Bar corresponds to 100 nm.

In both instances, microscopic analysis revealed spherical, single-layered (unilamellar) vesicles, consistent with previous results for TE of the same composition (Esposito et al., 2022). This specific structural organization could likely occur because the bulky hydrophilic head group of TW80 may provide steric hindrance, thereby reducing lamellar stacking between adjacent bilayers (Mkam Tsengam et al., 2022; Wongsirojkul et al., 2025). Finally, the average vesicle diameters aligned perfectly with the PCS measurements.

### 3.2 Stability studies

The stability of TE and TE_KF_ was assessed after 3 months of storage at 4 °C in the dark. PCS data demonstrated robust size stability for both formulations, with the Z-average and PDI remaining almost unchanged (Table 3). While the KF content remained highly stable, the Z-potential of the vesicles tended to fluctuate toward more negative values after 90 days of storage. This modest increase in negative surface charge is likely related to trace hydrolysis of PC ester bonds over prolonged aqueous storage, generating minor amounts of negatively charged free fatty acids, as well as gradual surface-rearrangement of ethanol and TW80 molecules at the lipid-water interface. Crucially, this increased negative charge enhanced inter-vesicular electrostatic repulsion, thereby helping to maintain the colloidal stability of the system without inducing vesicle aggregation or drug leakage.

**TABLE 3:** Physicochemical parameters of TE and TE_KF_ analyzed 1 day (d_0_) and 3 months (d_90_) after preparation.

Formulation		Z-Average (nm) ± s.d.	PDI ± s.d	Z-potential (mv) ± s.d.	KF content (mg/mL) ± s.d.
TE	d_0_	107.5 ± 1.82	0.13 ± 0.01	-23.80 ± 6.34	-
	d_90_	116.53 ± 4.08	0.16 ± 0.02	-27.05 ± 6.97	-
TE_KF_	d_0_	108.33 ± 3.85	0.14 ± 0.03	-20.21 ± 1.47	0.25 ± 0.03
	d_90_	113.86 ± 3.78	0.12 ± 0.05	-28.27 ± 4.72	0.25 ± 0.02

sd, standard deviation; data are the mean of 4 independent determinations on different batches.

### 3.3 Transethosomal gel preparation and characterization

To allow cutaneous application of TE, POL was employed as a thickening agent. Namely, TE gels were prepared by direct addition of POL (15%–20%, w/w) to TE. Homogeneous whitish formulations were obtained, with decreasing spreadability and leakage, inversely related to POL concentration (Table 4). Representative images of the leakage assay are reported in Supplementary Figure S3. Physical stability of the gel, evaluated by maintaining the formulations at 25 °C for 1 month, enabled the selection of a POL concentration of 20% w/w, since lower concentrations led to phase separation due to sedimentation.

**TABLE 4:** Percentage composition, spreadability and leakage values of TE-PG.

Formulation	PC	EtOH	TW80	POL	H_2_O	Spreadability^a^ (g cm/s)	Leakage^b^ (cm)
TE-PG_15_	0.77	24.47	0.26	15.0	59.5	23.17 ± 0.76	2.77 ± 0.25
TE-PG_18_	0.74	23.61	0.25	18.0	57.4	21.83 ± 0.58	0.33 ± 0.15
TE-PG_20_	0.72	23.04	0.24	20.0	56.0	18.67 ± 1.26	0.13 ± 0.05

^a^ Calculated as reported in Section 2.12

^b^ Running distance along the slide; data are the mean of 3 independent measurements ± s.d.

Thus TE-PG_20_, was selected for further studies and named TE-PG., The thickening of TE_KF_, with POL, 20% w/w resulted in TE_KF_-PG, a whitish and homogeneous gel with no differences with respect to TE-PG.

A rheological analysis was performed to evaluate the viscosity of TE-PG and TE_KF_-PG. Analyses were performed at 20 °C for flow ramps and oscillatory amplitude sweep (Figures 2A–D), and between 5 °C and 50 °C for temperature sweeps (Figures 2E,F).

**FIGURE 2:**
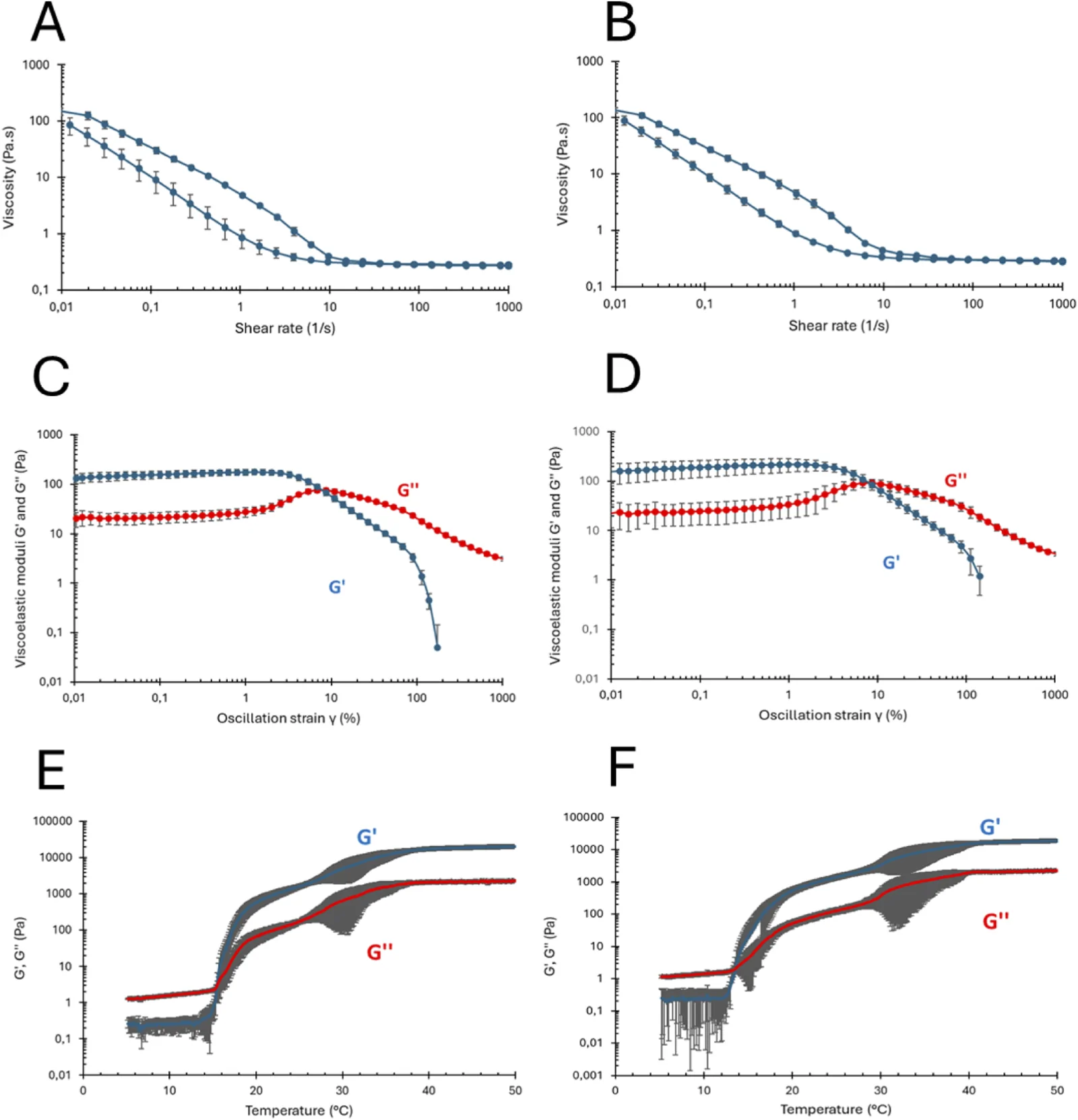
Rheological behavior of TE-PG **(A,C,F)** and TE_KF_-PG **(B,D,F)**. Flow ramps **(A,B)** and oscillatory amplitude sweep **(C,D)** were measured at T = 20 °C; temperature sweeps **(E,F)** were measured between 5 and 50 °C.

During flow ramps, both TE-PG (Figure 2A) and TE_KF_-PG (Figure 2B) exhibited shear-thinning behavior at low shear rates (up to 10 s^-1^), followed by a second Newtonian plateau at higher shear rates, indicating that the formulations had reached maximum structural breakdown. Additionally, thixotropic behavior was observed at low shear rates within the shear-thinning region, demonstrating a dependence on the shear history of the sample. In oscillatory amplitude sweeps, TE-PG (Figure 2C) and TE_KF_-PG (Figure 2D) displayed a linear viscoelastic region between 0.01% and 1% strain, where the storage modulus (*G′*) exceeded the loss modulus (*G″*). At strains above 10%, *G″* surpassed *G′* due to the structural breakdown of the samples. At last, in oscillatory temperature ramps, the elastic and viscous moduli *G′* and *G″* of TE-PG (Figure 2E) and TE_KF_-PG (Figure 2F) increased with temperature above 15 °C, with *G′* higher than *G″*, both moduli reaching high values. At low temperatures (below 15 °C, without KF and below 12 °C with KF), the formulations were more liquid than solid, with low values (about 1 Pa or lower). This demonstrates the strong evolution with temperature of the gel formulations, in the absence or in the presence of KF.

The presence of KF did not significantly change the rheological behavior in flow ramps (Figure 2B) or in oscillatory amplitude sweeps (Figure 2D), where the crossover stress (i.e., the stress value for which both moduli *G′* and *G″* are equal) was 8 ± 2 Pa for TE-PG and 10 ± 3 Pa for TE_KF_-PG. In contrast, in oscillatory temperature ramps, the presence of KF seemed to slightly decrease this critical temperature, from 15 °C without KF to 12 °C with KF (Figure 2F).

### 3.4 Saxs results

To gain further insight into the internal supramolecular organization of the formulations, SAXS measurements were performed on TE, TE_KF_, TE-PG and TE_KF_-PG samples. Measurements were carried out at 25 °C and 32 °C to compare the structural organization at room temperature and under physiological conditions. The corresponding SAXS profiles are shown in Figure 3.

**FIGURE 3:**
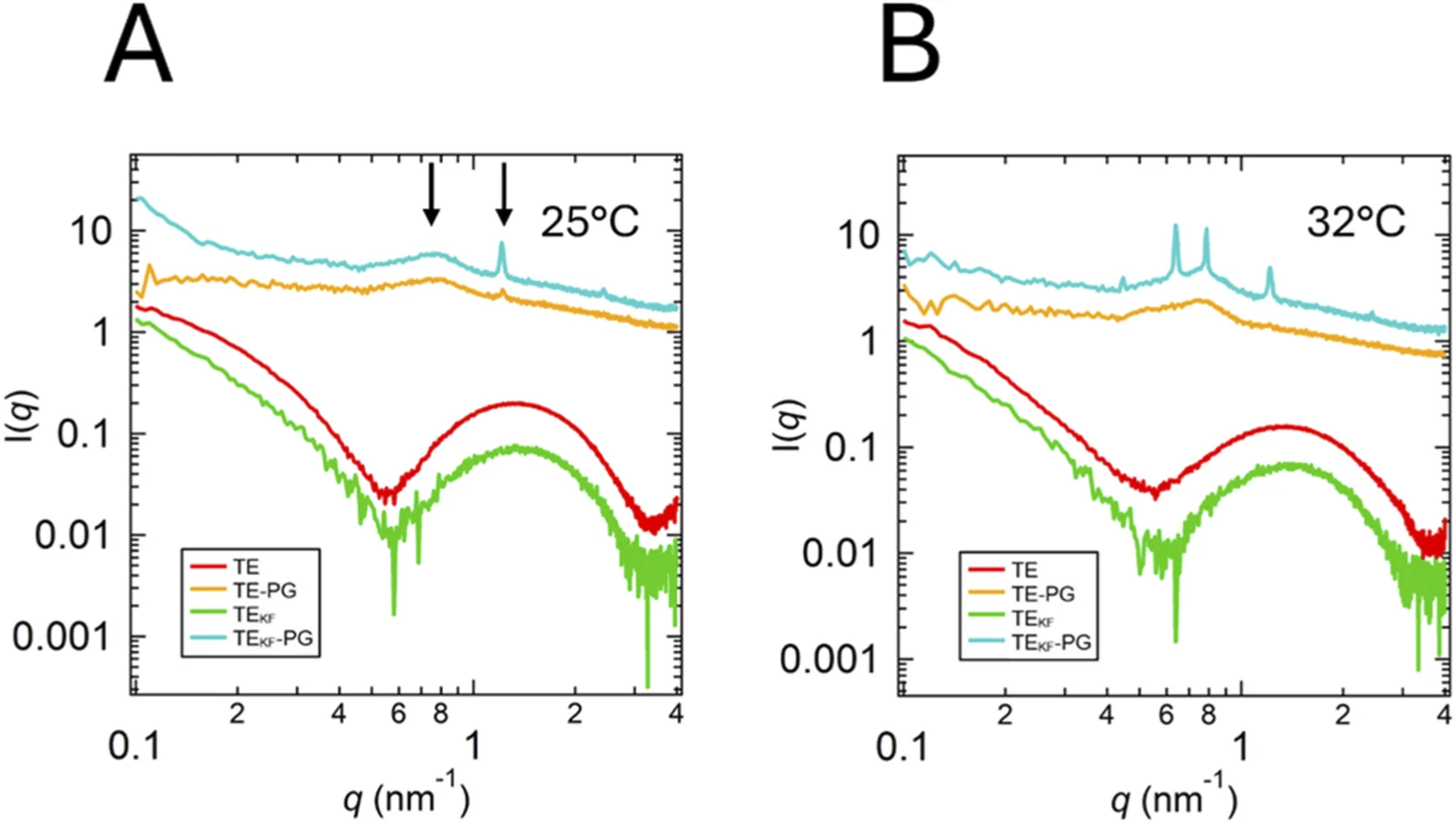
SAXS profiles of TE, TE_KF_, TE-PG and TE_KF_-PG, as indicated in the legend. Measurements were performed at 25 °C **(A)**, and 32 °C **(B)**. In each panel, the curves were scaled for clarity.

In agreement with Cryo-EM, SAXS analysis confirms that TE forms unilamellar lipid vesicles, as indicated by a broad scattering maximum over the q-range of approximately 0.6–3 nm^-1^. This feature arises from the form factor of the lipid bilayer and is consistent with a membrane thickness of about 4 nm. The incorporation of KF into TE (TE_KF_) does not appreciably alter the main SAXS features associated with the bilayer organization, indicating that the vesicular architecture is preserved. Moreover, the SAXS curves recorded at 25 °C and 32 °C are essentially unchanged, supporting the structural stability of both TE and TE_KF_ over this temperature range. At 32 °C, the two formulations show nearly superimposable profiles, with only minor differences in the low-q region suggesting slight variations in vesicle size distribution. The absence of Bragg reflections in all profiles further confirms the lack of long-range ordering within the vesicle dispersion.

A markedly different behaviour is observed after incorporation of the thickening agent POL (TE-PG). In this case, the scattering is no longer dominated by the vesicle bilayer form factor, but by the self-assembled POL structures, which largely mask the characteristic TE profile. A broad maximum centered at q≈0.7 nm^-1^ becomes the common and dominant feature of the SAXS curves at both temperatures, consistent with the scattering from POL micelles. In addition, at 25 °C a weak Bragg reflection appears at *q* = 1.22 nm^-1^, suggesting the onset of limited long-range ordering within the POL-rich domains. The black arrows in Figure 3 indicate these significant features. The addition of KF further enhances this structural ordering. At 25 °C, the intensity of the Bragg reflection increases and a second peak, consistent with the expected $1:(\sqrt{3)}:\sqrt{4}$ reflection sequence of a hexagonal phase, becomes detectable, indicating that KF promotes the ordering of the Poloxamer assemblies already present in the TE-PG formulation. Upon heating to 32 °C, the weak hexagonal order observed for TE-PG almost disappears, as indicated by the strong reduction of the corresponding Bragg reflection. In contrast, the TE_KF_-PG formulation undergoes a pronounced structural reorganization, with the appearance of three additional Bragg reflections at $q=0.454,0.639,\text{and }0.789\;nm^{-1}$ . Their reciprocal-space positions follow the characteristic sequence $1:\sqrt{2}:\sqrt{3}$ , which can be indexed as the first reflections of a cubic Pm $\underline{3}$ n phase (space group No. 223), corresponding to an ordered micellar cubic structure. These observations indicate that KF promotes a temperature-induced ordering of the POL-rich domains, leading to the coexistence of ordered lyotropic mesophases. Overall, the SAXS results suggest that KF reinforces the incipient hexagonal organization at room temperature and stabilizes a temperature-driven transition toward a highly ordered cubic structure under physiological conditions.

### 3.5 IVPT

To predict how TE vesicles and TE gel influence KF skin diffusion, Franz cells equipped with STRAT-M® membranes were utilized. Specifically, TE_KF_ and TE_KF_-PG were compared against a KF suspension in a 30:70 EtOH/H_2_O mixture (SUSP_KF_), with formulations maintaining a KF concentration of 0.25 mg/mL. The passive diffusion of a drug through a membrane or skin depends on two primary factors: (i) its partition coefficient between the vehicle and the membrane/skin, and (ii) its permeability coefficient (Kp) which is driven by the physicochemical properties of both the drug and the vehicle (Tapfumaneyi et al., 2025). The resulting KF diffusion profiles are illustrated in Figure 4, and the corresponding IVPT parameters are summarized in Table 5.

**FIGURE 4:**
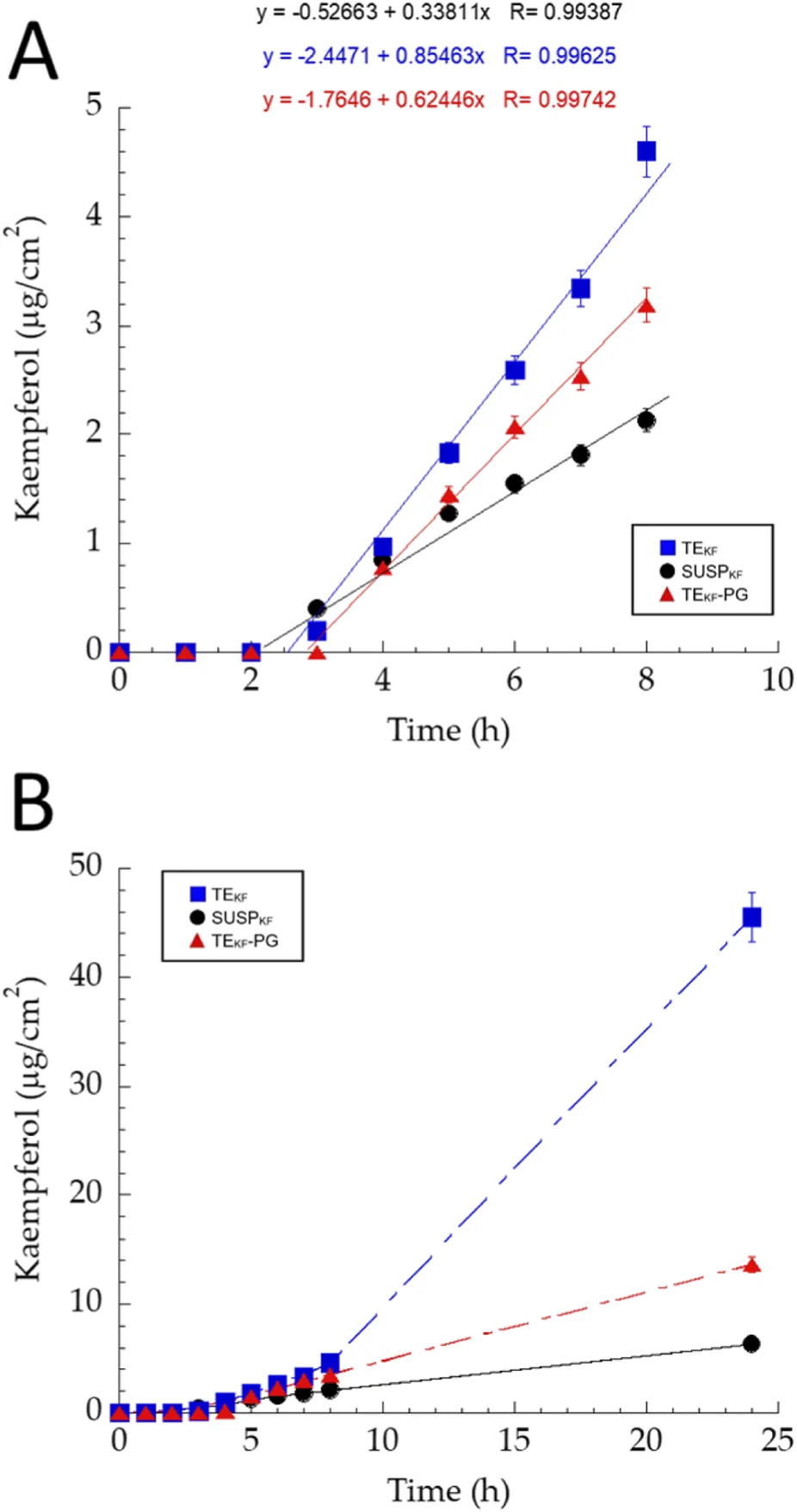
KF diffusion kinetics from TE_KF_ (blue squares), SUSP_KF_ (black circles) and TE_KF_-PG (red triangles), as determined by Franz cells associated with the Strat-M*®* membrane. **(A)** linear part of the kinetic, **(B)** profiles over 0–24 h. Data are the mean of 4 independent experiments ± s.d.

**TABLE 5:** IVPT parameters of the indicated formulations.

IVPT parameters	TE_KF_	SUSP_KF_	TEKF-PG
Jss^a^ (μg/cm^b^/h)	0.85 ± 0.15	0.34 ± 0.11	0.62 ± 0.01
Kp^b^ (cm/h) × 10^c^	3.4 ± 0.6	1.36 ± 0.44	2.48 ± 0.04
Tlag^c^ (h)	2.78 ± 0.82	2.21 ± 0.42	2.83 ± 0.74
A^d^ (μg/cm^b^)	45.51 ± 2.77	6.31 ± 1.88	13.61 ± 2.13

^a^ Steady-state flux per unit area.

^b^ Permeability coefficient.

^c^ Lag time.

^d^ Cumulative amount of KF, diffused at 24 h; KF, concentration was always 0.25 mg/mL. Data are the mean of 4 independent Franz cell experiments ± s.d.

KF diffusion through the membrane was faster in the case of TE_KF_ followed by TE_KF_-PG and SUSP_KF_. A lag time was observed, slightly longer in the case of TE_KF_-PG. The Kp value in the case of TE_KF_-PG was 1.82-fold higher than that of SUSP_KF_, and 1.37-fold lower with respect to TE_KF_, suggesting the vesicle transdermal potential. Notably, the amount of KF diffused after 24 h (A value) in the case of TE_KF_-PG was 2.15-fold higher than that of SUSP_KF_, and 3.34-fold lower with respect to TE_KF_.

### 3.6 Effect of kaempferol-loaded transethosome gel on human skin explants

The protective potential of TE_KF_-PG was evaluated *ex vivo* on human skin explants exposed to UV. In particular, the level of 4-HNE protein adducts (a marker of lipid peroxidation and protein oxidative damage) and COL I (collagen type I, a major structural component of the dermal extracellular matrix) (Ivarsson et al., 2025; Ivarsson et al., 2023) was evaluated by immunofluorescence at two time points: after a single UV exposure and formulation application (Day 1), and after three consecutive daily UV exposures and formulation applications (Day 3).

Figure 5 shows immunofluorescence images of skin samples labelled for 4-HNE, while signal quantification is reported in Figure 6. Briefly, at Day 1 (Figure 6A), skin samples exposed to UV or treated with SUSP_KF_ or TE prior to UV exposure all showed levels of 4-HNE higher than CT. Conversely, skin samples treated with TE_KF_-PG or TE_KF_ prior UV exposure showed levels of 4-HNE similar to CT. No significant difference between TE_KF_-PG and TE_KF_ was found (p = 0.230). At Day 3 (Figure 6B), all treated samples showed values of 4-HNE significantly higher than CT.

**FIGURE 5:**
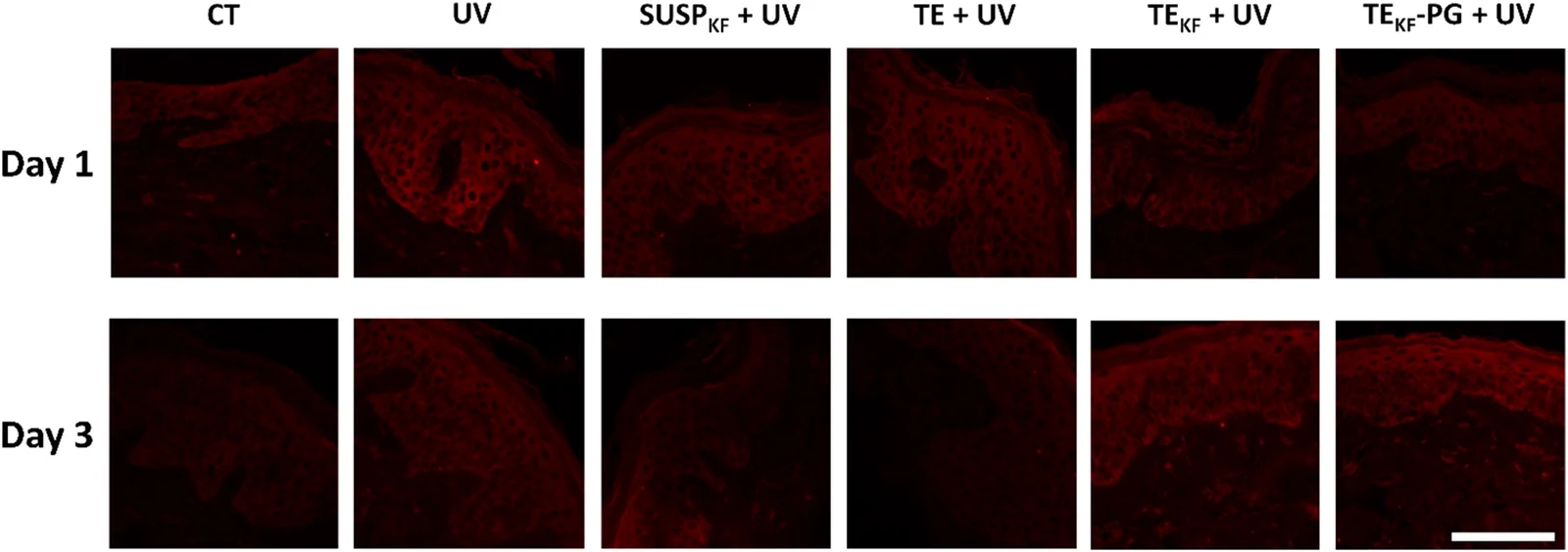
Immunofluorescence micrographs of human skin samples labelled for 4-HNE (red signal). Bar, 100 µm.

**FIGURE 6:**
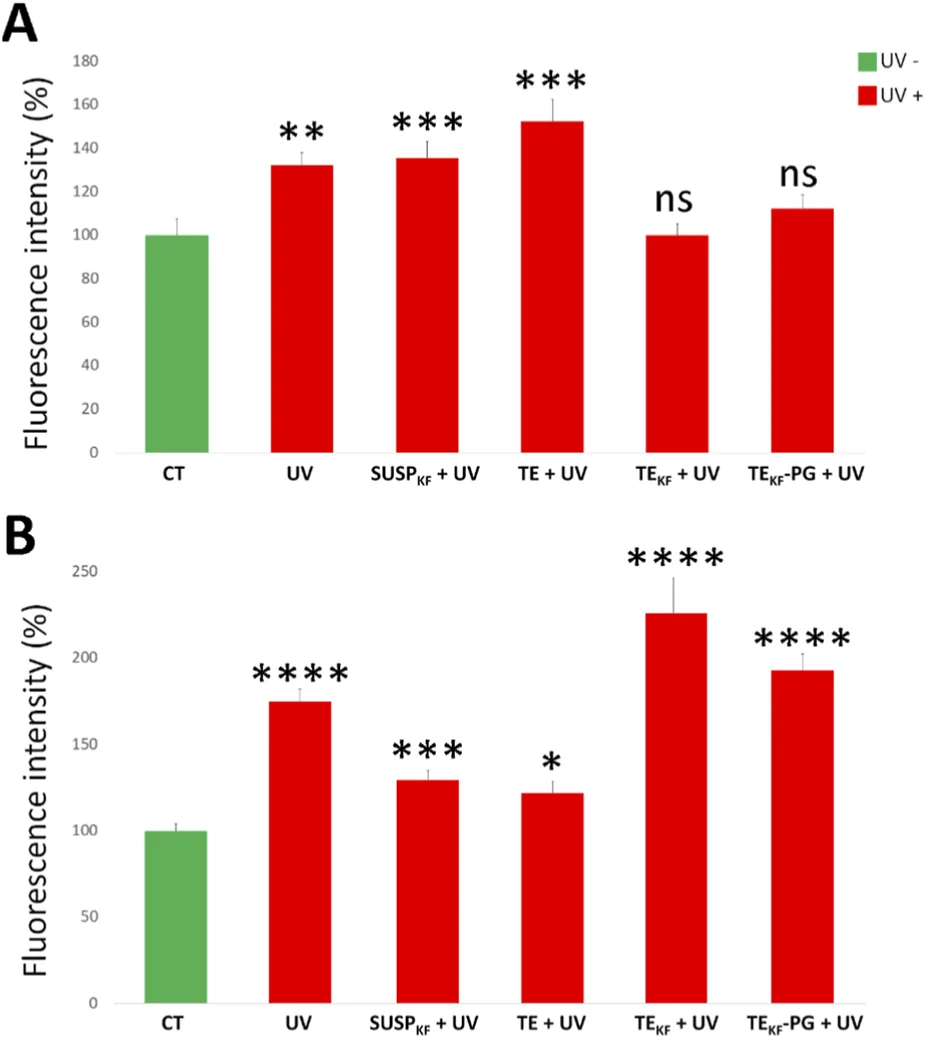
Mean values ± SE of anti-4-HNE fluorescence intensity in human skin samples at Day 1 **(A)** and Day 3 **(B)**. Statistical significance is represented as ns for p > 0.05, ∗ for p ≤ 0.05, ∗∗ for p ≤ 0.01, *** for p ≤ 0.001 and **** for p ≤ 0.0001.

Figure 7 shows immunofluorescence images of skin samples labelled for COL I; signal quantification is reported in Figure 8. Briefly, at Day 1 (Figure 8A) all treated samples showed values of COL I significantly lower than CT. At Day 3 (Figure 8B), skin samples exposed to UV or treated with TE prior exposure to UV showed levels of COL I significantly lower than CT. Conversely, skin samples treated with SUSP_KF_, TE_KF_-PG or TE_KF_ prior UV exposure showed levels of COL I similar to CT. No significant difference between TE_KF_-PG vs. TE_KF_ or vs. SUSP_KF_ was found (p = 0.892 and p = 0.088, respectively).

**FIGURE 7:**
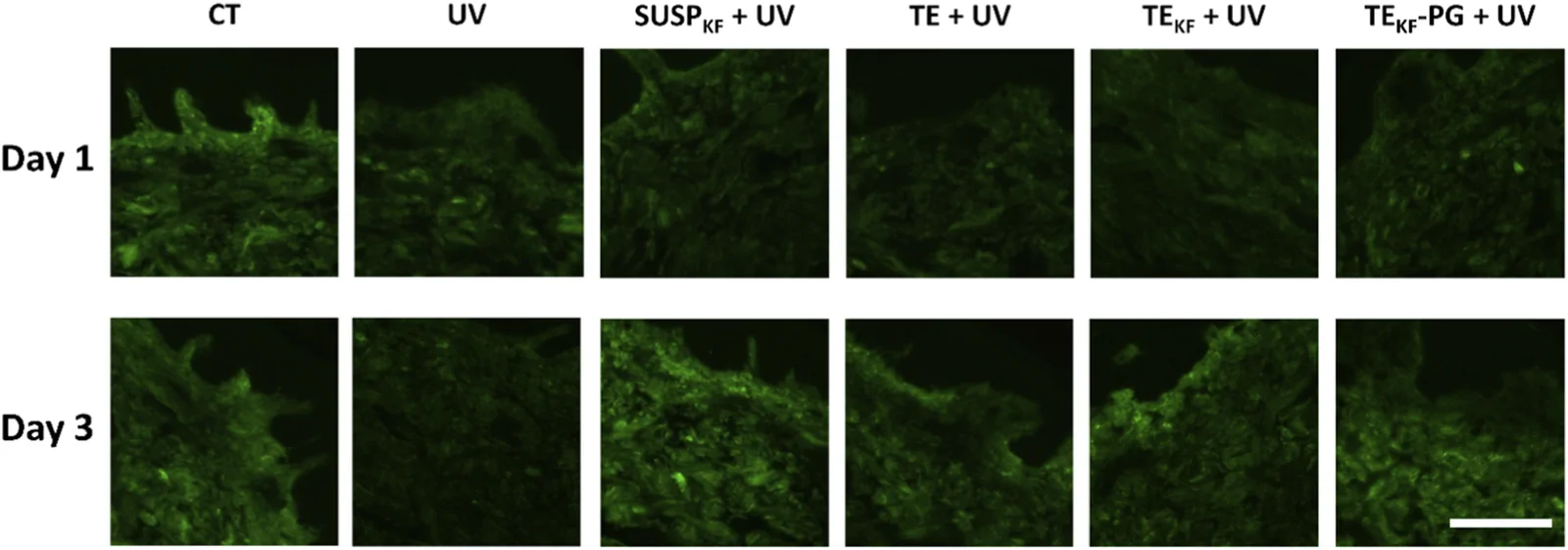
Immunofluorescence micrographs of human skin samples labelled for COL I (green signal). Bar, 100 µm.

**FIGURE 8:**
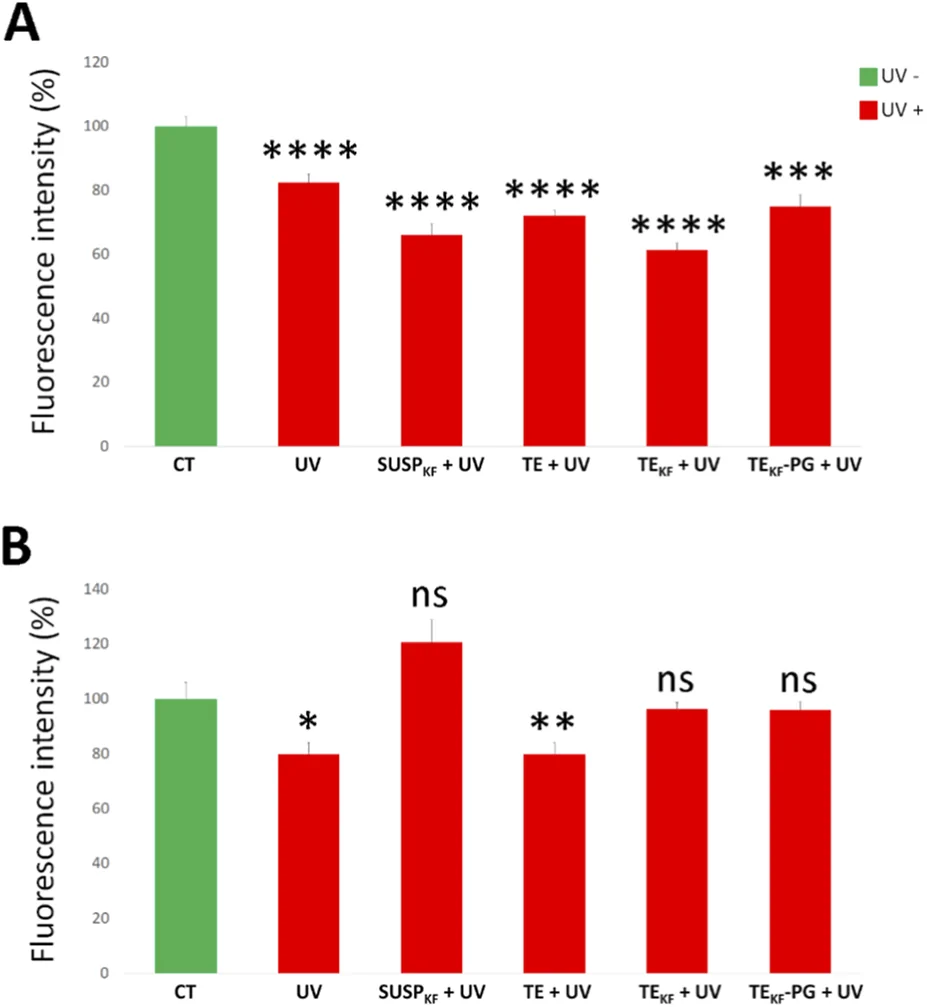
Mean values ± SE of anti-COL I fluorescence intensity in human skin samples at Day 1 **(A)** and Day 3 **(B)**. Statistical significance is represented as ns for p > 0.05, ∗ for p ≤ 0.05, ∗∗ for p ≤ 0.01, *** for p ≤ 0.001 and **** for p ≤ 0.0001.

## 4 Discussion

The preformulation study demonstrated the successful development of TE_KF_-PG as a stable topical delivery vehicle for KF. While standard ET underwent phase separation upon KF loading, the inclusion of TW80 as an edge activator yielded high EC (> 96%), uniform and stable unilamellar vesicles. The entrapment of KF into TE slightly modulated the vesicular surface charge without compromising colloidal stability. As both TE and TE_KF_ share identical vehicle compositions, this variation in Zeta potential confirms the localization and active interaction of KF within the vesicular membrane. The intercalation of the lipophilic flavonol into the PC bilayer likely promotes hydrogen bonding with the polar headgroups, reorienting membrane dipoles and partially screening surface charges at the vesicle-water interface. The higher TE vesicle stability with respect to ET could be attributed to the presence of TW80, characterized by an unsaturated alkyl chain along with sorbitan and PEG moieties, enabling stabilization of the PC bilayer architecture (Wongsirojkul et al., 2025). To address the liquid nature of TE formulations and prevent skin runoff, thickening TE with 20% w/w POL imparted a shear-thinning, thermo-responsive gel matrix that transitions into a structured network above low temperatures. SAXS analysis revealed that KF actively promotes supramolecular ordering within the poloxamer domains, stabilizing a transition into a highly ordered cubic Pm 3 n mesophase at skin physiological temperature (32 °C). Ultimately, IVPT studies using Strat-M® membranes confirmed that TE_KF_-PG significantly improves KF flux and permeability over a simple drug suspension, striking an optimal balance between matrix-controlled sustained release and enhanced transdermal penetration. Overall, TE_KF_-PG provides a dual-control platform for topical KF delivery. While liquid TE_KF_ exhibit higher initial *in vitro* skin flux owing to the absence of a polymer barrier, their low viscosity limits clinical applicability due to rapid runoff from the application site. The hydrogel matrix in TE_KF_-PG imparts essential rheological properties for topical administration, extending local skin residence time and yielding a sustained release profile.

The evaluation of formulation effectiveness in protecting skin from UV damage was performed *ex vivo* on human skin explants. In fact, unlike the *in vitro* model used for IVPT, this *ex vivo* model shows the structural complexity of the human skin, with all its cell types, extracellular matrix components, and appendages, maintaining the ability to respond to stimuli similarly to *in vivo* (although for a limited time related to skin preservation). The *ex vivo* study demonstrated that the tested TE_KF_-PG formulation can limit the oxidative damage induced by UV exposure. In fact, the application of TE_KF_-PG as well as of TE_KF_ on the *stratum corneum* before UV exposure prevented the rise of 4-HNE observed in UV-treated samples and in samples treated with both UV and TE (therefore in the absence of KF). 4-HNE is a highly reactive and cytotoxic molecule produced by lipid peroxidation and triggers a cascade of pro-inflammatory signaling pathways (Atalay Ekiner et al., 2024). This finding is therefore consistent with the previously reported antioxidant properties of KF (Sharma et al., 2021; Alrumaihi et al., 2024). However, our findings demonstrated that SUSP_KF_ was unable to prevent the formation of 4-HNE in the skin, highlighting the key role of nanovesicles to ensure the effectiveness of KF in transdermal applications, likely facilitating the crossing of the *stratum corneum* barrier, as previously demonstrated (Esposito et al., 2022). Interestingly, TE_KF_-PG prevented 4-HNE accumulation similarly to TE_KF_, demonstrating that the gelling process does not decrease the effectiveness of the nanoformulation.

However, the antioxidant effect of TE_KF_-PG and TE_KF_ proved to be limited. In fact, both TE_KF_-PG and TE_KF_ exerted their protective antioxidant action 24 h after a single UV exposure, as indicated by the lack of 4-HNE increase at Day 1, but both formulations were unable to limit 4-HNE production after repeated UV treatments for three consecutive days, as demonstrated by the high levels found at Day 3. It is likely that this challenging UV treatment caused a severe oxidative damage in the skin, and the tested nanoformulations could not prevent the rise of 4-HNE levels produced by lipid peroxidation, even through repeated applications.

UV-induced oxidative stress is also responsible for the degradation and loss of type I collagen in skin, probably through activation of MMP-2 (Ivarsson et al., 2025). Accordingly, in this work we found that, after 24 h from UV exposure, all skin samples contained lower amounts of type I collagen in comparison to CT, irrespective of the application of any formulation. However, after 3 days of repeated UV exposures and formulation applications, all KF formulations (SUSP_KF_, TE_KF_ and TE_KF_-PG) proved to restore type I collagen levels like CT. Conversely, in skin treated with UV or UV + TE (i.e., in the absence of KF) the collagen levels remained significantly lower than CT. This strongly suggests that KF is able to exert cutaneous anti-degenerative effects in the longer term, maybe by inhibiting MMPs and/or stimulating collagen synthesis as reported in other biological systems (Lin et al., 2013; Salehi Namini et al., 2025; Sugita et al., 2026). Our findings demonstrated that this restorative effect is similar for all KF formulations and is not hampered by the loading in TE, nor by their gelling process.

Although the present study did not evaluate specific markers of collagen synthesis or direct indices of collagen degradation, and COL1 immunofluorescence does not directly measure collagen degradation, our findings indicate that UV exposure leads to COL1 loss. Finally, the differences observed between Day 1 and Day 3 likely reflect the evolving net balance among UV-induced matrix damage, collagen turnover, tissue remodeling, and recovery. Unfortunately, in this work, it was not possible to understand the contribution of each process.

Recently, Fang et al. (Fang et al., 2025) evaluated a liposomal gel loaded with KF for topical treatment of acute eczema, demonstrating strong *in vitro* and *in vivo* antioxidant activity, as well as promising therapeutic efficacy. Although the KF liposomal hydrogel demonstrated good homogeneity and stability during preparation, its high viscosity could hinder uniform topical application and compromise patient acceptability in clinical settings. Rather than using Carbopol as the thickening agent, as reported by Fang et al., we incorporated the thermoreversible polymer POL, selecting its concentration based on spreadability and leakage evaluations. This formulative assessment led to the selection of 20% (w/w) POL, yielding TE_KF_-PG with a texture and viscosity optimal for cutaneous administration.

## 5 Conclusion

In conclusion, this study demonstrates that the TE_KF_-PG could serve as a novel hydrogel-integrated vesicular system. By combining the skin-penetration capability of TE with the structural viscosity and bioadhesion of a gel network, this formulation offers a balanced approach for localized, sustained topical drug delivery. *Ex vivo* assessments on human skin explants revealed a distinct, time-dependent photoprotective profile: while the nanoformulation was essential to deliver KF and acutely prevent UV-induced lipid peroxidation (suppressing 4-HNE accumulation after 24 h), long-term repeated treatment effectively restored type I collagen levels, preventing matrix degradation likely via the modulation of MMPs. Importantly, the loading of KF in TE_KF_-PG preserved the drug functional biological activity. Together, these findings highlight TE_KF_-PG as a promising, multi-functional therapeutic platform to combat acute UV-induced oxidative damage and long-term dermal photoaging.

## Data Availability

The original contributions presented in the study are included in the article/Supplementary Material, further inquiries can be directed to the corresponding authors.
