# Supplementary material for: Formulation and ex vivo characterization of a kaempferol transethosomal hydrogel for the prevention of UV-induced cutaneous damage
Source: Front Bioeng Biotechnol. 2026 Oct 12;14:1963532. doi: 10.3389/fbioe.2026.1963532 (PMC13696495; doi:10.3389/fbioe.2026.1963532)
Supplement: Supplementary file 1 [file DataSheet1.pdf]

Supplementary Materials

Formulation and Ex-Vivo Characterization of a Kaempferol Transethosome Gel with Anti-Inflammatory Potential.

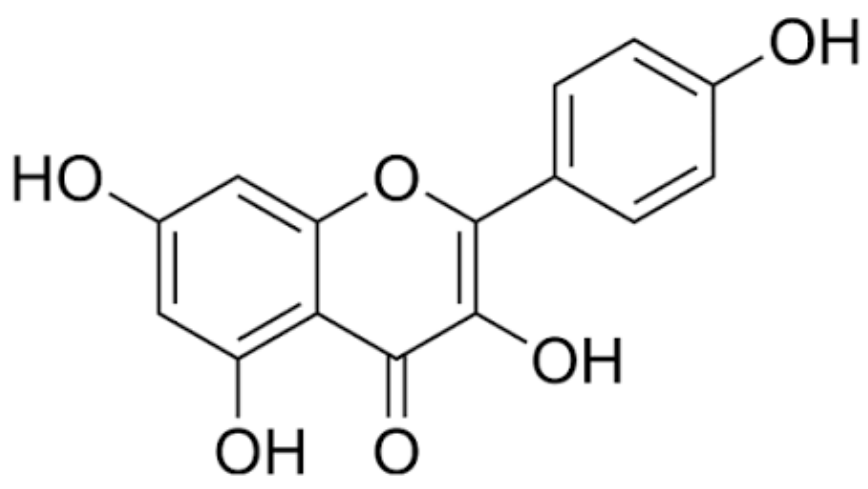

Figure S1 *Kaempferol Chemical structure.*

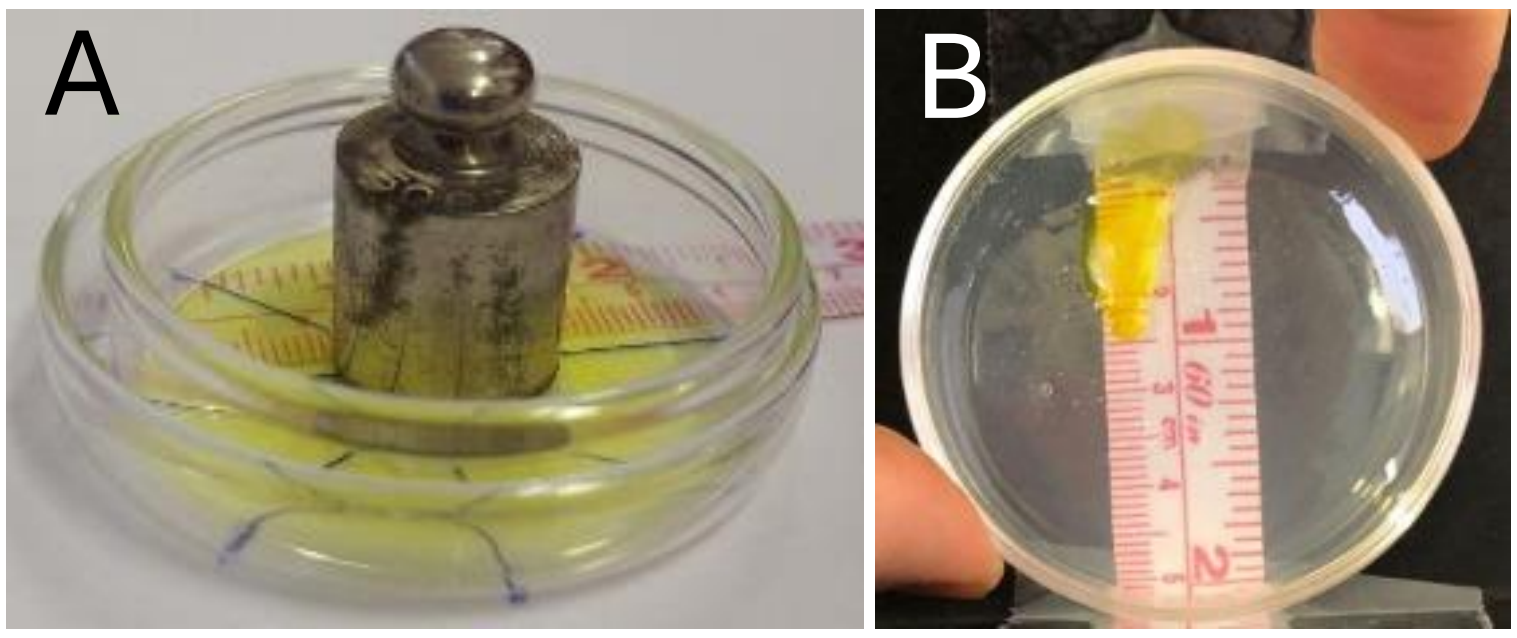

Figure S2 *Spreadability (A) and Leakage (B) assay setup.*

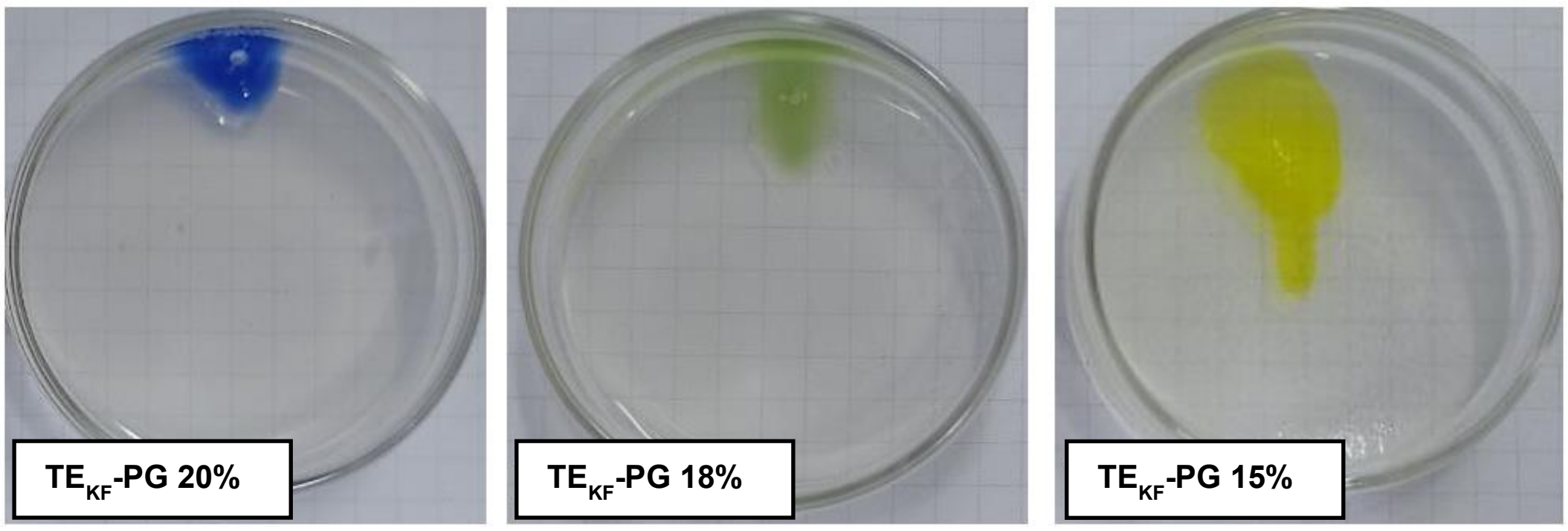

Figure S3 *Leakage assay representative images.*
